# Retraction: Overexpression of Cosmc suppresses cell migration and invasion in different subtypes of breast cancer cells via Tn and T glycans

**DOI:** 10.1042/BSR-2019-1062_RET

**Published:** 2022-09-21

**Keywords:** Apoptosis, Breast cancer, Cosmc, Metastasis, Proliferation, Tn and T glycans

This article is being retracted from *Bioscience Reports* at the request of the authors following receipt of a notification from a reader alerting the Editorial Office to multiple concerns surrounding the validity of the results and conclusions of this paper, specifically regarding the flow cytometric analysis. A reader on Pubpeer has also identified a duplication across papers between the oe-Cosmc/MDA-MB-453-Merge panel of figure 3C and the merged pLV-EGFP-N panel of figure 3B in an article by Xu et al. (doi: 10.1007/s13402-019-00490-8). The authors have also stated that since to publishing the paper, they have discovered that the original cells used were contaminated and the results and conclusions are therefore not reliable. The authors have been unable to replicate the results and wish to retract the article. The Editor-in-Chief and Editorial Board agree with the Retraction.

